# Intermittent Hypoxia-Induced Cognitive Deficits Are Mediated by NADPH Oxidase Activity in a Murine Model of Sleep Apnea

**DOI:** 10.1371/journal.pone.0019847

**Published:** 2011-05-23

**Authors:** Deepti Nair, Ehab A. Dayyat, Shelley X. Zhang, Yang Wang, David Gozal

**Affiliations:** Department of Pediatrics, Pritzker School of Medicine, University of Chicago, Chicago, Illinois, United States of America; Louisiana State University Health Sciences Center, United States of America

## Abstract

**Background:**

In rodents, exposure to intermittent hypoxia (IH), a hallmark of obstructive sleep apnea (OSA), is associated with neurobehavioral impairments, increased apoptosis in the hippocampus and cortex, as well as increased oxidant stress and inflammation. Excessive NADPH oxidase activity may play a role in IH-induced CNS dysfunction.

**Methods and Findings:**

The effect of IH during light period on two forms of spatial learning in the water maze and well as markers of oxidative stress was assessed in mice lacking NADPH oxidase activity (*gp91phox* ^_*/Y*^) and wild-type littermates. On a standard place training task, *gp91phox* ^_*/Y*^ displayed normal learning, and were protected from the spatial learning deficits observed in wild-type littermates exposed to IH. Moreover, anxiety levels were increased in wild-type mice exposed to IH as compared to room air (RA) controls, while no changes emerged in *gp91phox* ^_*/Y*^ mice. Additionally, wild-type mice, but not *gp91phox* ^_*/Y*^ mice had significantly elevated levels of NADPH oxidase expression and activity, as well as MDA and 8-OHDG in cortical and hippocampal lysates following IH exposures.

**Conclusions:**

The oxidative stress responses and neurobehavioral impairments induced by IH during sleep are mediated, at least in part, by excessive NADPH oxidase activity, and thus pharmacological agents targeting NADPH oxidase may provide a therapeutic strategy in sleep-disordered breathing.

## Introduction

Obstructive Sleep Apnea (OSA), a clinical syndrome characterized by repeated episodes of upper airway obstruction during sleep, is now recognized as a significant and highly prevalent health problem, due not only to its cardiovascular and metabolic morbidity, but also because of the prominent cognitive and behavioral implications of the disease. The neuropsychological impairments are accompanied by increased levels of systemic markers of oxidative stress and inflammation in addition to gray matter loss in neural sites contributing to cognitive function [1]–[5]. The inordinate sensitivity of neuronal tissues to alterations in oxygen homeostasis has led to the hypothesis that the behavioral consequences and cellular losses observed in OSA patients are produced, at least in part, by the episodic hypoxia-reoxygenation cycles during sleep that characterize OSA. In support of this hypothesis, rodent models have demonstrated that chronic exposure to intermittent hypoxia during the rest period (IH), in the absence of significant sleep fragmentation, is accompanied by neurodegenerative changes, increased oxidant stress and inflammation, and impaired spatial learning in the Morris water maze [6]–[18], and that genetic or pharmacological manipulations of oxidative stress pathways attenuated IH-induced deficits [18], [19].

NADPH oxidase has been primarily studied in the context of its role in phagocyte oxidative burst [20]. This enzyme has however emerged as a major source of ROS generation in mammalian cells, including the CNS [21]–[24], and a role for NADPH oxidase in astrocyte function has been described [25]. Moreover, it has been demonstrated that NADPH oxidase is expressed in neurons [26], [23] and localized at synapses [26]. NADPH oxidase is composed of two membrane-bound subunits (gp91*phox* and p22*phox*) and three cytosolic subunits, which include p47*phox*, p67*phox*, and Rac [27]. The membrane-bound subunits form a heterodimer that stabilizes them within the membrane, whereas the cytosolic subunits are recruited to the membrane following stimulation. Complete complex assembly is necessary for full NADPH oxidase activity [28]. Mutations in the gp91*phox* and p47*phox* genes are the most common mutations that cause chronic granulomatous disease (CGD; [29]). These mutations disable the NADPH oxidase complex, thereby preventing the oxidation of NADPH and the subsequent production of superoxide [30], [31], which is required for pathogen destruction as well as most superoxide-dependent signal transduction in nonphagocytic cells [27], [32], [33]. gp91*phox* [34] and p47*phox* [35] mutant mice have been generated and can therefore be used to explore the putative role of NADPH oxidase in murine models of sleep apnea.

In the present study, we examined this hypothesis in NADPH oxidase mutant mice (gp91*^phox-/Y^*) and wild type littermates by exposing them to IH, and assessed changes in hippocampus-dependent learning and memory tasks. In addition, we also tested other behavioral paradigms for anxiety and depression, since such problems are frequently encountered in patients with sleep apnea [36]–[41].

## Materials and Methods

### Animals

**Figure 1:**
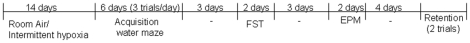
Schematic diagram on the sequence of behavioral experiments and exposures to either IH or RA in both wild type and NADPH oxidase knock-out mice.

Male hemizygous *gp91phox-^/Y^* (B6.129S-*Cybb^tm1Din^*/J) mice (20–22 grams) and C57BL/6J mice (20–22 grams) were purchased from Jackson Laboratories (Bar Harbor, Maine), housed in a 12 hr light/dark cycle (lights on from 7:00 am to 7:00 pm) at a constant temperature (26±1°C). Mice were housed in groups of four in standard clear polycarbonate cages, and were allowed access to food and water *ad libitum*. All behavioral experiments were performed during the light period (between 9:00 am and 12:30 pm). Mice were randomly assigned to either IH or room air (RA) exposures. The experimental protocols were approved by the Institutional Animal Use and Care Committee and are in close agreement with the National Institutes of Health *Guide in the Care and Use of*. All efforts were made to minimize animal suffering and to reduce the number of animals used. Figure 1 summarizes the timeline of the treatments and the behavioral experiments. After each behavioral paradigm the mice were immediately returned back to their respective exposures.

### Intermittent Hypoxia Exposures

Animals were maintained in 4 identical commercially-designed chambers (30″x20″x20″; Oxycycler model A44XO, BioSpherix, Redfield, NY) operated under a 12 hour light-dark cycle (7:00 am–7:00 pm) for 14 days prior to behavioral testing. Oxygen concentration was continuously measured by an O_2_ analyzer, and was changed by a computerized system controlling gas outlets, as previously described [6], [42], [43], such as to generate oxyhemoglobin nadir values in the 65–72% range every 180 seconds. Ambient temperature was kept at 22–24^°^C.

### Behavioral Testing

The Morris water maze was used to assess spatial reference learning and memory, as well as working memory. The maze protocol is similar to that described by Morris [44] with modifications for mice. The maze consisted of a white circular pool, 1.4 m in diameter and 0.6 m in height, filled to a level of 35 cm with water maintained at a temperature of 21°C (Morris 1984). Pool water was made opaque by addition of 150 ml of non-toxic white tempera paint. A Plexiglas escape platform (10 cm in diameter) was positioned 1 cm below the water surface and placed at various locations throughout the pool. Extramaze cues surrounding the maze were located at fixed locations, and visible to the mice while in the maze. Maze performance was recorded by a video camera suspended above the maze and interfaced with a video tracking system (HVS Imaging, Hampton, UK).

Briefly, a standard place-training reference memory task was conducted on mice in the water maze following exposure to 14 days of IH or RA. One day prior to place learning, mice were habituated to the water maze during a free swim. Place learning was then assessed over six consecutive days using a spaced training regimen that has been demonstrated to produce optimal learning in mice [45]. Each training session consisted of three trials separated by a 10 minute inter-trial interval (ITI). On a given daily session, each mouse was placed into the pool from 1 of 4 quasirandom start points (N, S, E or W) and allowed a maximum of 90 seconds to escape to the platform where the mice were allowed to stay for 15 sec. Mice that failed to escape were led to the platform. The position of the platform remained constant during the trials. 24 h following the final training session, the platform was removed for a probe trial to obtain measures of spatial bias. To assess the performance in the water maze, mean escape latencies and swim distance were analyzed.

#### Reference memory

Retention tests were carried out 14 days after acquisition of the task. In the retention test, performance in a single session (two trials) was assessed, and the mean average performance of the two trials was calculated.

#### Elevated plus maze (EPM)

The elevated plus maze (EPM) was used to assess anxiety. The basic measure is the animal preference for dark, enclosed places over bright, exposed places [46]. A 60 w light was placed above the apparatus and the test was video taped by an overhead camera. Mice were placed in the center of the maze facing a closed arm, and allowed to explore for 10 min in isolation. Each mouse received one videotaped trial. Mice prefer to enter into closed arms compared to open arms. Time spent in the dark area is viewed as avoidance or anxiety-like behavior. The following parameters were scored: (a) Percent time spent in open and closed arms; (b) number of entries to closed arms; (c) Time spent in the center. An arm entry was defined as the entry of all four feet into either one of the closed arm. Of note, the maze was cleaned with 30% ethanol between trials to remove any odor cues.

#### Forced swimming test (FST)

Briefly, mice were individually forced to swim in an open cylindrical container (diameter 14 cm, height 20 cm), with a depth of 15 cm of water at 25±1 °C. The immobility time, defined as the absence of escape-oriented behaviors, was scored during 6 min, as previously described [47], [48], [49]. Each mouse was judged to be immobile when it ceased struggling, and remained floating motionless in the water, making only those movements necessary to keep its head above water. The average percentage immobility was calculated by a blinded experimenter.

#### NADPH Oxidase Expression

qRT-PCR analysis of p47phox was performed using ABI PRISM 7500 System (Applied Biosystems, Foster City, CA). RNA from frontal cortex in IH and RA exposed mice was prepared with TRIZOL. cDNA synthesis was performed using a High-Capacity cDNA Archive Kit (Applied Biosystems, Foster City, CA). Ribosomal 18S rRNA was used as a reference gene to normalize the expression ratios for the gene of interest. Primer sequences were 5′-CAGCCAGCACTATGTGTACA-3′ and 5′-GAACTCGTAGATCTCGGTGAA-3′ for p47phox (91 bp). One microgram of total RNA was used to generate cDNA templates and TaqMan® Master Mix Reagent Kit (Applied Biosystems, Foster City, CA) was used to amplify and quantify the p47phox transcript in 25 µl reactions. Triplicate PCR reactions were performed in 96-well in parallel with the 18S rRNA. The steps involved in the reaction program included: the initial step of 2 minutes at 50°C; denaturation at 95°C for 10 min, followed by 45 thermal cycles of denaturation (15 seconds at 95°C) and elongation (1 min at 60°C). Expression values were obtained from the cycle number (Ct value) using the Biosystems analysis software. P47phox and 18S rRNA were performed in triplicates to determine the Ct-diff. These Ct values were averaged and the difference between the 18S Ct (Avg) and the gene of interest Ct (Avg) was calculated (Ct-diff). The relative expression p47phox was analyzed using the 2^-ΔΔCT^ method. Quantitative results were expressed as the mean ±standard deviation (SD). Statistical significance was evaluated by the Student's t-test.

#### Measurement of NADPH oxidase activity

NADPH oxidase activity was measured using the standard cytochrome c reduction method, as described previously [50]. Isolated frontal cortices from wild type mice exposed to either IH or RA were homogenized in a RIPA buffer. The homogenate was subjected to centrifugation at 250 g for 10 min to remove cellular debris. Supernatant was then centrifuged at 20,000 g for 20 min at 4 °C to eliminate mitochondria, lysosomes, peroxisomes, Golgi membranes, and rough endoplasmic reticulum. The resulting supernatant was centrifuged at 100,000 g for 60 min at 4°C. The pelleted plasma membrane fraction containing Nox was dissolved in a buffer containing 8 mM piperazine-N,N'-bis 2-ethanesulfonic acid (pH 7.2), 100 mM KCl, 3 mM NaCl, 3.5 mM MgCl2, 1.25 mM EGTA, and proteolytic inhibitors. The solubilized membrane fraction samples were placed in a multiwell plate, and flavin adenine dinucleotide (FAD; 0.01 mM), acetylated cytochrome c (0.1 mM), and GTPγS (0.01 mM, as an activating agent for NADPH oxidase) were added to the samples. Superoxide dismutase (SOD; 100 U/ml) was included to block cytochrome c reduction in half of the samples. After the samples were kept at room temperature for 2 min, sodium dodecyl sulfate (0.1 mM) as an additional activating agent was added. After incubation for 3 min, NADPH (0.2 mM) was added to initiate cytochrome c reduction. Absorbance at 550 nm (A550) in samples incubated and not incubated with SOD was measured for 10 min on a FlexStation III microplate reader (Molecular Devices, Sunnyvale, CA, USA). NOX activity was calculated as the SOD-inhibitable reduction of cytochrome c. All chemicals were from Sigma (St. Louis, MO).

#### Lipid Peroxidation Assay

MDA-586 kits (OxisResearch, Portland OR) were used to measure the relative malondialdehyde (MDA) production, a commonly used indicator of lipid peroxidation (50), in frontal brain cortex according to the manufacturer's instructions. Briefly, after anesthesia with pentobarbital (50 mg/kg intraperitoneally), mice were perfused with 0.9% saline buffer for 5 minutes and the cortex was dissected, snap frozen in liquid nitrogen, and stored at –80°C until assay the following day. Cortical tissues were homogenized in 20 mM phosphate buffer (pH 7.4) containing 0.5 mM butylated hydroxytoluene to prevent sample oxidation. After protein concentration measurements, equal amounts of proteins (2.0–2.5 mg protein from each sample) were used in triplicate to react with chromogenic reagents at 45°C in 500 µL buffer for 1 to 2 hours. The samples were then centrifuged and clear supernatants measured at 586 nm. The level of MDA production was then calculated with the standard curve obtained from the kit according to the manufacturer's instructions.

#### 8-hydroxydeoxyguanosine (8-OHDG)

Levels of 8-OHDG were measured in frontal brain cortex using a commercially available assay (Cell Biolabs, San Diego, CA). Briefly, cortical samples or 8-OHDG standards were first added to an 8-OHDG/BSA conjugate preabsorbed enzyme immunoassay plate. After a brief incubation, an anti–8-OHDG mAb was added, followed by an horseradish peroxidase-conjugated secondary antibody. The 8-OHDG content in the cortical samples was then determined by comparison with the 8-OHDG standard curve.

#### Data Analysis

To elucidate the nature of interactions between IH and RA conditions, all data were analyzed by one way ANOVA. First, overall statistical significance was determined for the entire training period between the treatment groups. In addition, two-way repeated measures ANOVA were used to analyze each trial blocks, followed by post-hoc Tukey tests. Similar statistical approaches were used to compare probe trial, reference memory, EPM and FST. For all comparisons, a p value <0.05 was considered to achieve statistical significance.

In all the experimental conditions, the data were divided into 6 blocks (containing 3 trials/day). We used a multivariate MANOVA model (SPSS software 11; Chicago) that included latency, pathlength and swim speed and Two between factors: (1) Groups (four levels): RA C57BL6J, IH C57BL6J, RA *gp91phox* ^_*/Y*^ and IH *gp91phox* ^_*/Y*^ (2) Condition (two levels): RA or IH. All *F* statistics are reported using Pillai's Trace. The interaction of three different factors, i.e., time, condition and group were determined using this mixed model repeated measures MANOVA.

## Results

### 

#### Spatial Learning Performance

**Figure 2:**
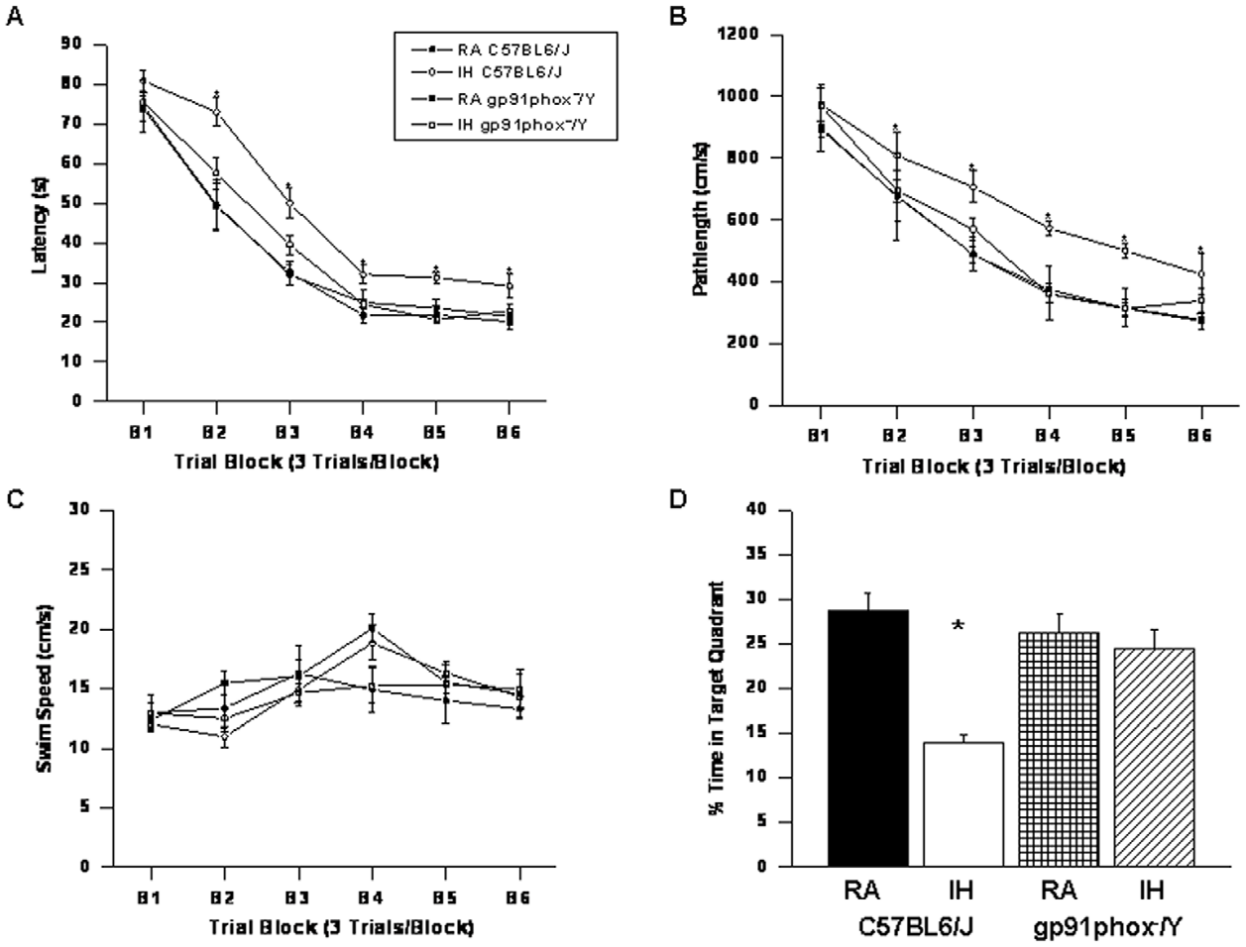
gp91phox^_/Y^ mice exposed to IH do not exhibit any deficits in learning and memory functions. (A and B) Mean latencies (s) and pathlengths (cm) to locate the target platform during place training in C57BL6/J and *gp91phox* ^_*/Y*^ either exposed to intermittent hypoxia (IH) or maintained in room air (RA) (n = 12 per group. (C) Swim Speed (*D*) Mean percentage time in the target quadrant during probe trial after completion of water maze testing in either C57BL6/J and *gp91phox* ^_*/Y*^ exposed to IH or maintained in RA. (n = 12/experimental group; *P<0.05).

**Figure 3:**
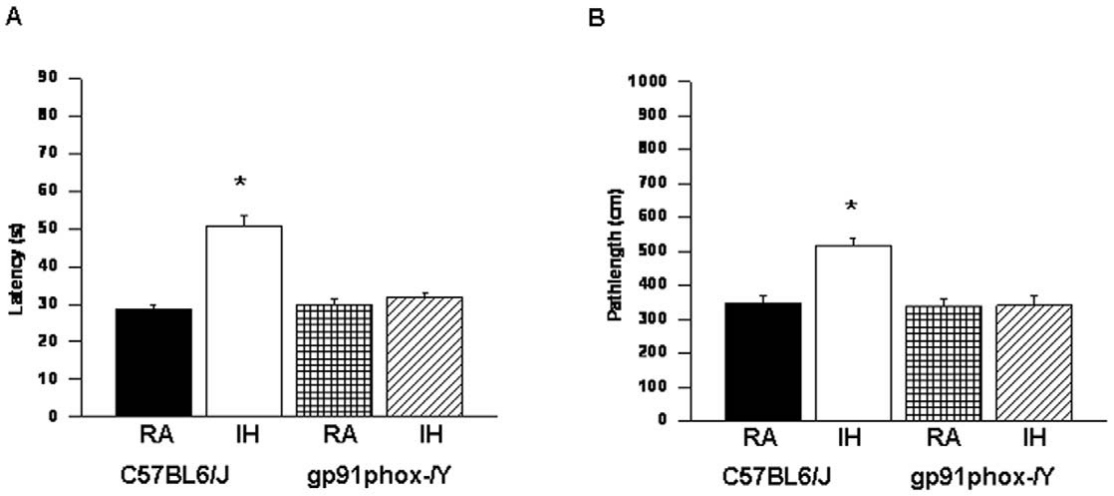
gp91phox^_/Y^ mice exposed to IH do not exhibit any deficits in retention. (A) Mean latencies (s) and (B) pathlengths (cm) to locate the target platform during retention in C57BL6/J and *gp91phox* ^_*/Y*^ either exposed to intermittent hypoxia (IH) or maintained in room air (RA) during retention of the Morris water maze task. (n = 12/experimental group; *P<0.05).

On a standard place discrimination task, wild type mice exposed to 14 days of IH (IH-C57BL6/J) exhibited longer latencies and pathlengths to locate the hidden platform when compared to room air controls RA-C57BL6/J, RA- *gp91phox* ^_*/Y*^ and *gp91phox* ^_*/Y*^ mice exposed to 14 days IH (IH- *gp91phox* ^_*/Y*^) animals (n = 12 per experimental condition; Figures 2A and B). Overall latency analysis for the entire trial blocks revealed significant changes between the different treatment groups, [F =  41.14; p<0.001] and pathlength, [F = 16.44; p<0.001] indicating that IH adversely affected task performance. Significant differences in latencies were observed during blocks 2 [F = 4.91; p<0.006], 3 [F = 8.38; p<0.001], 4 [F = 3.35; p<0.03], 5 [F = 7.06; p<0.001] and 6 [F = 4.457; p<0.01]. There were no significant differences in Block 1. Repeated measures ANOVA revealed significant differences in pathlengths during blocks 3 [F = 5.25; p<0.004], 4 [F = 4.36; p<0.01], 5 [F = 6.73; p<0.001] and 6 [F = 2.99; p<0.04], with no significant differences in Blocks 1 and 2. There were no significant differences in swim speed in these mice. In the probe-trial test, one-way ANOVA revealed a significant effect of treatment [IH vs. RA: F = 12.87; p<0.001]. The magnitude of impairment was greatest in IH-C57BL6/J mice (Figure 2C). In the reference memory tests, IH-C57BL6/J mice exhibited significant deficits in memory retention in both latency [F = 19.61; p<0.001] and pathlength [F = 12.84; p<0.001]. However, the IH- *gp91phox* ^_*/Y*^ mice performed similar to normoxic controls (Figure 3).

Repeated measures MANOVA with latency, groups and conditions [F_(5,39)_ = 72.54; *P*<0.0001]; revealed that RA *gp91phox* ^_*/Y*^ and RA C57BL6/J mice required significantly less time than their littermates exposed to IH to find the hidden platform in a Morris water maze (Figure 2A); Repeated measures MANOVA with pathlength, groups and conditions [F_(5,39)_  = 21.409; *P*<0.0001]; indicated that as the training progressed the RA *gp91phox* ^_*/Y*^ and RA C57BL6/J mice could reach the hidden platform and covered the shortest distance when compared to the distance covered by their littermates exposed to IH in a Morris water maze (Figure 2B). In addition repeated measures MANOVA with swim speed, groups and conditions on the swim speed showed no significant differences between the groups and treatments (Figure 2C).

### Elevated Plus Maze

**Figure 4:**
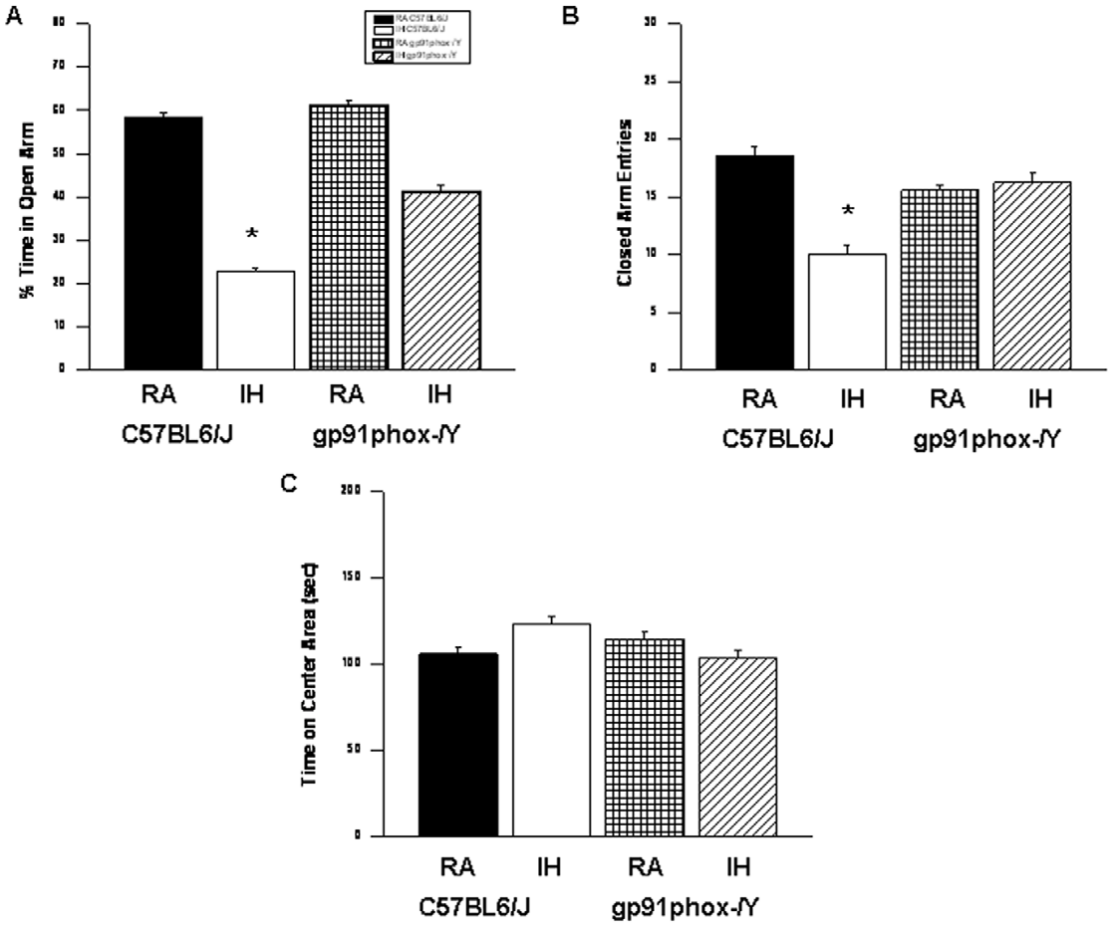
Exposure to IH induces anxiety in mice. C57BL6/J mice exposed to IH spend significantly less time in the open arm of the elevated plus maze compared to RA C57BL6/J, or *gp91phox* ^_*/Y*^ mice exposed to either RA or IH (*A*). A reduced number of closed-arm entries emerged in wild type mice exposed to IH (*B*). (C) Time spend in the Center Area was increased in wild type mice exposed to IH (n = 12/experimental group; *P<0.05).

IH-C57BL6/J mice showed significant differences in the percentage of time spent in the open arm [F = 64.13; p<0.001] and in the number of entries into the closed arm [F = 14.74; p<0.001] (Figure 4). The results of the elevated plus maze showed that IH-C57BL6J spent significantly less time in the open arms (Figure 4; group effect, [F =  18.354; p<0.001] and significantly more time in the center area (Figure 4; group effect, [F =  26.945; p<0.001]. The number of entries into the closed arms was significantly increased (Figure 4; condition effect, [F =  11.533; p<0.001].

Although, the percentage of time spent in the open arm is commonly used as a measure of anxiety, the time spent on the center platform of the maze and the closed arm entries all reflect anxiety-like behaviors in mice. [79], [87].

#### Forced Swim Test

**Figure 5:**
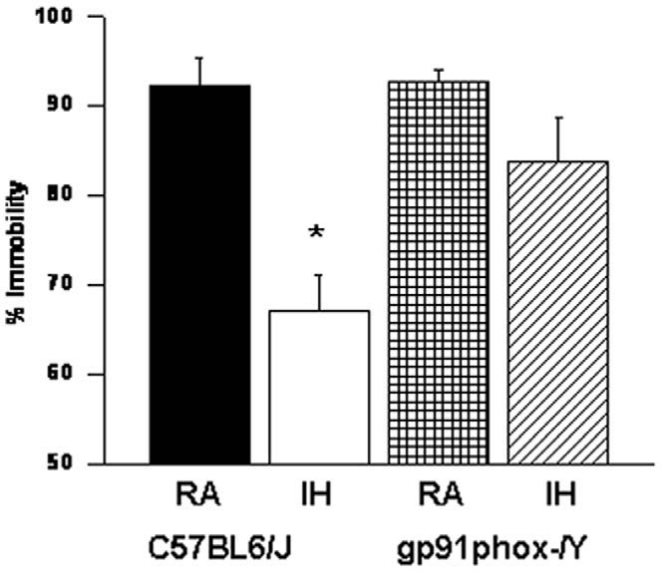
Forced-swim test indicates gp91phox^_/Y^ mice are not depressed following IH. *gp91phox* ^_*/Y*^ exposed to IH show less immobility as compared to C57BL6/J mice exposed to IH. *P<0.05. See text for more details.

IH-C57BL6/J mice had significantly higher immobility durations during the last 4 min of the FST [F = 25.54; p<0.001] when compared to all other treatment groups, including IH- *gp91phox* ^_*/Y*^ (Figure 5).

#### NADPH Oxidase Expression and Activity

**Figure 6:**
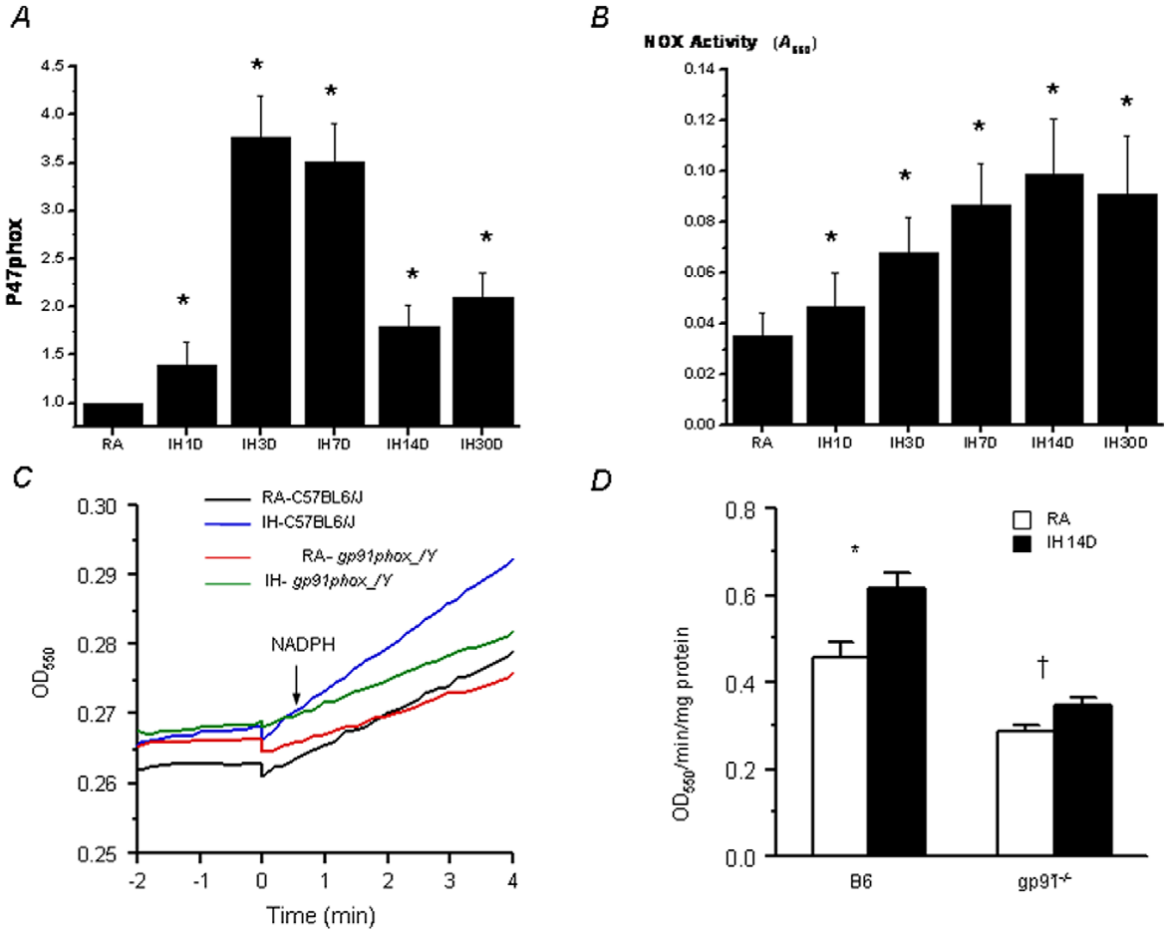
Changes in NADPH oxidase expression and activity. (A). Changes in P47phox mRNA expression in frontal cerebral cortex in wild type mice exposed to IH (n = 8 per experimental group; *P<0.05). (B). Changes in NADPH oxidase activity in frontal cerebral cortex in wild type mice exposed to IH (n = 8 per experimental group; *P<0.05). (C) Kinetic NADPH oxidase activities in hippocampus measured as NADPH-dependent cytochrome c reduction. Shown are representative tracings from the four experimental groups. (D) Summary of NADPH oxidase activities in hippocampus. IH resulted in a substantial increase in NOX activities in wild type mice (*P = 0.002). Such IH-induced increases of NADPH oxidase activity were much attenuated in *gp91phox* ^_*/Y*^ mice, although the increase was still significant (†P = 0.024; n = 6 for each group).

Frontal cortical tissues from IH- and RA-exposed mice were subjected to quantitative RT-PCR. P47phox expression was increased in IH starting at 3 days and sustained thereafter (Figure 6a). Similarly, NADPH oxidase activity was significantly increased in IH-exposed wild type mice (Figure 6b and 6d). Furthermore, such IH-induced increases of NOX activity were attenuated in gp91^-/-^ mice, although the increase was still significant (p = 0.024; n = 6/group; Figure 6c and 6d).

#### Lipid Peroxidation

**Figure 7:**
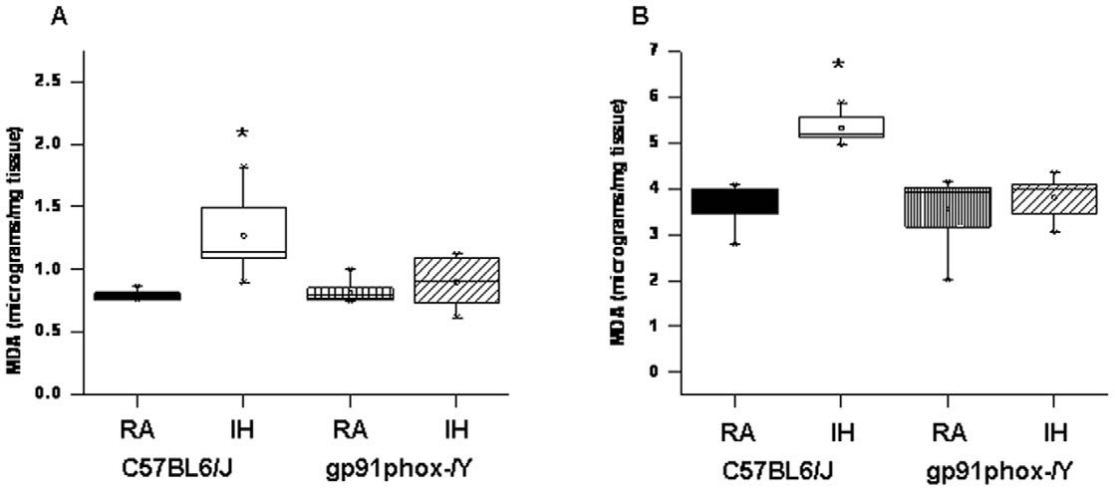
Lipid peroxidation was reduced in the frontal cortex of gp91phox^_/Y^ exposed to IH. MDA tissue levels in cortex and hippocampus of *gp91phox* ^_*/Y*^ and C57BL6/J mice exposed to either room air (RA) or intermittent hypoxia for 14 days (IH). (n = 6 per experimental group; *P<0.05).

After the behavioral experiments, cortical tissues and hippocampus were harvested and processed for assessment of lipid peroxidation as indicated by MDA levels. Figure 7 shows MDA concentrations in homogenates of cerebral cortex from all treatment groups. A significant increase in MDA levels was observed in IH-C57BL6/J mice [F = 10.38; p<0.001] in the cortex and [F = 35.416; p<0.001] in the hippocampus when compared to all other groups.

#### 8-OHDG Levels

**Figure 8:**
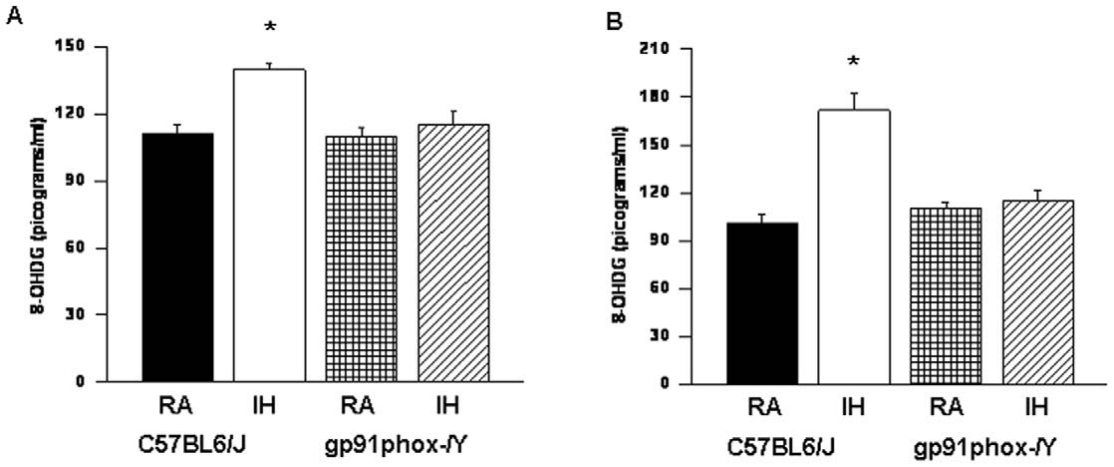
Oxidative DNA damage was present in the cortex of gp91phox^_/Y^ exposed to IH. 8-OHDG levels in cortex and hippocampus of *gp91phox* ^_*/Y*^ and C57BL6/J mice exposed to either room air (RA) or intermittent hypoxia for 14 days (IH). (n = 6 per experimental group; *P<0.05).

The levels of 8-OHDG in homogenates of cerebral cortex and the hippocampus were significantly higher in IH-C57BL6/J mice [F = 32.50; p<0.001] and [F = 22.214; p<0.001] respectively; when compared to all other groups (Figure 8). However there were no significant differences in the levels of 8-OHDG in cortex of IH- *gp91phox* ^_*/Y*^ when compared to RA controls.

## Discussion

OSA is a clinical condition that is now recognized as having important neurobehavioral consequences that stem, at least in part, from pathological inflammatory processes that are associated with the disease [51]–[53]. During sleep, patients with OSA undergo repeated periods of IH, which essentially correspond to recurring episodes of hypoxia and reoxygenation, leading to increased levels of ROS that are considered as contributing to end-organ injury, including the CNS [54]–[57]. In the present study, we provide conclusive results suggesting that increased activity of NADPH oxidase-mediated oxidative stress pathways mediates, at least in part the effects of IH on cognitive and behavioral functions. Reactive oxygen species (ROS) can be generated from various subcellular compartments, including mitochondria, the cellular membrane, lysosomes, peroxisomes, and the endoplasmic reticulum [24], [58]–[61]. For example, NADPH oxidase [24], [62], [63], xanthine oxidase [63], phospholipase A2 [64], lipoxygenases and cyclooxygenase [58], and cytochrome P450 [65] have all been identified as sources of ROS in various subcellular compartments under both physiological and pathological conditions. Although NADPH oxidase is primarily expressed in phagocytic cells, increasing evidence suggests that various subunits of NADPH oxidase are also expressed in nonphagocytic cells such as sympathetic ganglion neurons and cortical neurons [66]–[68], thereby supporting the conceptual framework that neurons in general express NADPH oxidase [69]. In this context, NADPH oxidase has been implicated in conditions that remotely resemble hypoxia-reoxygenation [70]–[76]. Activation of NADPH oxidase leads to generation of the superoxide ion (O^−^_2_), a ROS which can be converted to the highly reactive hydroxyl radical and to peroxynitrite, a highly damaging RNS [77], [78]. To date, five NADPH oxidase enzyme (NOX) isoforms have been identified (NOX 1–5), and localization studies have shown that of the five NOX enzymes, the NOX2 and NOX4 isoforms are highly localized in the hippocampus CA1 and cerebral cortex [79], [80]. Our study provides insight into the functional role of NADPH oxidase activation and its contribution to intermittent hypoxia induced cognitive deficits. Our studies in the Morris Water Maze revealed preservation of spatial learning and memory in *gp91phox* ^_*/Y*^ mice as compared to the wild type mice after exposures to intermittent hypoxia. Furthermore, swim speed was not different between the groups, demonstrating that the differences were not due to differences in locomotor ability or coordination. The Morris Water Maze is a hippocampal-dependent test of learning and memory, and thus the enhanced performance on the Morris Water Maze of the *gp91phox* ^_*/Y*^ mice after IH, but not in normoxic conditions, is likely reflective of the attenuation of oxidative stress and enhanced neuronal survival in the hippocampus.

As would be anticipated from such considerations, we indeed found that IH exposures markedly increased the expression and activity of NADPH oxidase in wild type mice, thereby confirming the assumption that conditions mimicking the oxygenation patterns of sleep apnea induce activation of NADPH oxidase in cortical brain regions. While we can not infer from our current findings on the cellular source of NADPH oxidase contribution to IH-induced cognitive and behavioral deficits, the near complete abrogation of such deficits in the *gp91phox* ^_*/Y*^ mice clearly and conclusively assigns a critical role for NADPH oxidase in this context. Moreover, NADPH oxidase inhibition is neuroprotective of hippocampus CA1 pyramidal cells 7 days after ischemia [81]–[83]. Of note, we have previously shown that the IH-induced cognitive and behavioral deficits are attenuated by reductions in oxidative stress and inflammatory signaling cascades through pharmacological interventions [9], [10], as well as through attenuation of oxidative stress via targeted genetic manipulations of manganese superoxide dismutase, platelet-activating factor receptor, or nitric oxide synthase [16], [18], [19].

Depressive and anxiety symptoms are frequent in OSA patients [40], [84], [85]. Moreover, depression may account for the fatigue seen in OSA patients, even after OSA severity has been controlled [38]. The prefrontal cortex is particularly vulnerable to sleep disruption, and hypoxemia further creates an unfavorable cellular environment for the restorative processes to occur [2]. The elevated plus-maze is the most frequently utilized animal model for assessing anxiety-like behaviors [86], [87] since it enables researchers to observe the conflict between two innate rodent behaviors, namely the avoidance of open space exposure as countering the tendency to explore novel environments [87]. Our results show that intermittent hypoxia modified anxiety-like behavior in wild type mice. In contrast, *gp91phox* ^_*/Y*^ exposed to intermittent hypoxia showed preserved performances in this test, suggesting that regions underlying these behavioral patterns are susceptible to IH, most likely via the oxidant stress mediated by activation of NADPH oxidase. Our findings are not surprising considering the previous reports on the palliative effect of apocynin, a putative NADPH oxidase antagonist, on neuronal viability in the context of intermittent hypoxia [88]. Furthermore, Veasey and collaborators further expanded on the critical role played by NADPH oxidase in the injury to locus coeruleus neurons and the excessive sleepiness that developed as a consequence of IH during sleep [89], [90].

The cumulative evidence supports the assumption that the structural and neurobehavioral consequences of IH exposures in adult rodents involve a number of interrelated pathways, namely glutamate excitoxicity, oxidative stress, mitochondrial dysfunction, up-regulation of pro-inflammatory mediators, and altered regulation of pro- and anti-apoptotic gene cascades [91]–[93]. However, the explicit mechanistic involvement of NADPH oxidase in the CNS end-organ injury was not thoroughly explored. We propose that activation of NADPH oxidase by IH would be lead to the observed increases in oxidative stress markers. Earlier findings from our laboratory showed that intermittent hypoxia increases NADPH oxidase subunit protein expression in hypoxia sensitive brain regions involved in learning and memory in rats, and that administration of green tea polyphenols in drinking water attenuated the increase in NADPH oxidase gene expression under IH conditions [94].

In summary, we have shown that excessive NADPH oxidase-mediated superoxide release induced by IH contributes to the cellular damage and consequent behavioral impairments associated with severe forms of OSA. This study suggests that NADPH oxidase may be a promising target for OSA treatment, especially in halting the progression of OSA-associated cognitive and behavioral morbidities.
